# Recycling of Expired Ceftamil Drug as Additive in the Copper and Nickel Electrodeposition from Acid Baths

**DOI:** 10.3390/ijerph18189476

**Published:** 2021-09-08

**Authors:** Delia-Andrada Duca, Mircea Laurențiu Dan, Nicolae Vaszilcsin

**Affiliations:** Faculty of Industrial Chemistry and Environmental Engineering, University Politehnica Timișoara, 300223 Timișoara, Romania; duca.delia@gmail.com (D.-A.D.); nicolae.vaszilcsin@upt.ro (N.V.)

**Keywords:** expired drug, drug recycling, electroplating additive, levelling agent, galvanotechnics, Tafel method, electrochemical impedance spectroscopy

## Abstract

Due to the large quantity of expired and unused drugs worldwide, pharmaceutical disposal has become a serious problem that requires increased attention. In the present paper, the study on recycling ceftazidime (CZ) as an additive in copper and nickel electrodeposition from acid baths is highlighted. CZ is the active substance from expired commercial drug Ceftamil^®^. Its electrochemical behavior was studied by cyclic voltammetry. As well, kinetic parameters for copper and nickel electrodeposition were determined using Tafel plots method at different temperatures and CZ concentrations in these acid baths. The activation energy was calculated from Arrhenius plots. Electrochemical impedance spectroscopy was used to investigate the charge transfer resistance and coverage degree in the electrolyte solutions at several potential values. Gibbs free energy values, calculated from Langmuir adsorption isotherms, revealed the chemical nature of CZ–electrode surface interactions. The favorable effect of the organic molecules added in copper and nickel electroplating baths was emphasized by optical microscope images. The morphology of the obtained deposits without and with 10^−4^ mol L^−1^ CZ was compared. The experimental results revealed that expired Ceftamil^®^ is suitable as additive in copper and nickel electroplating processes from acid baths.

## 1. Introduction

Every year, a great amount of pharmaceutical products expires around the world, making them unusable for the treatment of patients or other medical purposes [1,2,3,4,5,6,7,8,9]. Most of them are incinerated in order to avoid environment contamination [10,11,12,13], but some of them, as in the household area, are difficult to track and commonly end up in the residual water due to improper disposal techniques [14,15,16,17]. Unfortunately, a large number of unused drugs have become hazardous contaminants in soil, surface and ground water, endangering ecosystem and human health [18,19].

Certain products from a large number of drugs contain only active substances, without excipients. These are usually organic molecules with a complex chemical structure and large molecular area, which can find other utilities in various electrochemical processes, like levelling or brightening agents in galvanotechnics [20,21,22], anodes for lithium-ion batteries [23], as well as corrosion inhibitors for metals in different aggressive media [24,25,26].

In terms of environmental impact, the possibility to reuse expired drugs as additives in galvanic baths is the most advantageous, because only a very small fraction of them is embedded in the final cathodic deposit. Electroplating bath composition remains constant for a long time, requiring corrections only at appreciable intervals [27,28,29]. Additives are very important due to their influence on physical and mechanical properties of the deposited metallic layer. Generally, they act by adsorption onto the metal surface, inhibiting the electrodeposition process, thus reducing the grain size of the metallic deposit [30,31,32].

**Figure 1:**
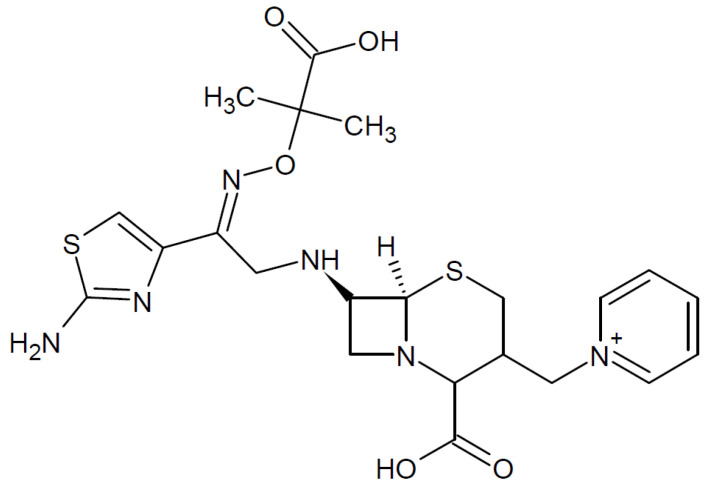
CZ chemical structure.

CZ is an antibiotic from third generation cephalosporins group with a large activity spectrum, but enhanced activity against Pseudomonas spp. The IUPAC name of CZ is: (Z)-(7R)-7-[2-(2-Aminothiazol-4-yl)-2-(l-carboxy-l-methylethoxyimino)acetamido]-3-(l-pyridiniomethyl)-3-cephem-4-carboxylate pentahydrate. Its chemical structure is presented in Figure 1.

CZ contains several structures, such as double bounds, aromatic rings, and heteroatoms with lone pair electrons which confer its inhibitive properties in the electrode processes [33].

In this paper, the possibility to use CZ active substance from expired Ceftamil^®^ drug as additive in copper and nickel electroplating is studied. A similar galvanic baths composition was used by Badarulzaman et al. [34] and Pasquale et al. [35] for nickel and copper electrodeposition, respectively.

## 2. Materials and Methods

Electrochemical measurements were performed using a PARSTAT 2273 potentiostat/galvanostat in a 150 mL thermostatted glass cell. Pt, Cu, and Ni electrodes, having 0.5 cm^2^ exposed area, were used as working electrodes, two graphite rods as counter electrodes and saturated Ag/AgCl as reference. All further potentials from the experimental work are referred to this electrode (*E*_Ag/AgCl_ = +0.197 V vs. normal hydrogen electrode).

CZ electrochemical behavior has been studied by cyclic voltammetry. Cyclic voltammograms (CVs) have been recorded on Pt electrode in 0.5 mol L^−1^ H_2_SO_4_ as well in 0.5 mol L^−1^ Na_2_SO_4_ + 30 g L^−1^ H_3_BO_3_ electrolyte solution (BS) in the absence and presence of different CZ concentrations, with scan rates between 5 and 500 mV s^−1^, at 25 °C temperature.

Kinetic parameters for copper and nickel electrodeposition have been calculated from Tafel plots. Linear voltammograms (LVs) have been drawn with low scan rate (2 mV s^−1^) in 25–65 °C temperature range on copper electrode in 5 g L^−1^ Cu^2+^ solution (0.5 mol L^−1^ H_2_SO_4_ + 5 g L^−1^ Cu^2+^ from CuSO_4_·5H_2_O) and on nickel electrode in 5 g L^−1^ Ni^2+^ solution (30 g L^−1^ H_3_BO_3_ + 5 g L^−1^ Ni^2+^ from 20.32 g L^−1^ NiSO_4_·7H_2_O and 3.05 g L^−1^ NiCl_2_·6H_2_O) in the absence and presence of different concentrations of CZ. For both processes, the activation energy has been calculated from Arrhenius plots.

Based on the dependence between the CZ concentration added in the electrolyte solutions and surface coverage degree, using Langmuir adsorption isotherms, drawn at different deposition potentials, Gibbs free energy values were calculated. From its values, the nature of interaction between CZ and the metal substrate can be appreciated.

Electrochemical impedance spectroscopy (EIS) studies were recorded on a BioLogic SP150 potentiostat/galvanostat equipped with an EIS module, in the frequency range between 10 mHz and 100 kHz, the amplitude of the alternating voltage was 10 mV. For each measurement 60 points with a logarithmic distribution of 10 points per decade were recorded. The experimental data were fitted using the ZView–Scribner Associates Inc. software and equivalent electrical circuits by applying the Levenberg–Marquardt least squares complex non-linear fitting algorithm. The EIS results are given in Supplementary Materials (Figures S3–S5, Tables S1 and S2).

Further, copper discs were used as substrate for copper and nickel electrodeposition. Copper was plated in a bath containing 0.5 mol L^−1^ H_2_SO_4_ and 250 g L^−1^ CuSO_4_·5H_2_O, while for nickel a Watts bath (300 g L^−1^ NiSO_4_·7H_2_O, 45 g L^−1^ NiCl_2_·6H_2_O and 30 g L^−1^ H_3_BO_3_) was used. Both depositions were performed at 25 °C from baths without and with 10^−4^ mol L^−1^ CZ. The morphology and roughness of the surfaces was evaluated using a laser microscope (Lext OLS 4000, 3D measuring laser microscope, Olympus). The results from the analysis gained by using the roughness measurement module from the microscope software were compared. The measured values were calculated on surfaces collected on micrographs taken at 100× magnification.

All test solutions were prepared from high purity reagents: H_2_SO_4_ Merck p.a. 95–97%, Na_2_SO_4_ anhydrous Sigma-Aldrich p.a. ≥99%, H_3_BO_3_ Sigma-Aldrich p.a. ≥99.5%, CuSO_4_·5H_2_O Merck p.a. ≥98%, NiSO_4_·7H_2_O Sigma-Aldrich p.a. ≥99%, NiCl_2_·6H_2_O Merck p.a. ≥97%. In the experimental studies, different concentrations of CZ, between 10^−6^ and 10^−3^ mol L^−1^, from expired Ceftamil^®^ commercial drug (expiration date: January 2013) were used as additive in the electrolyte solutions. A vial of Ceftamil^®^ contains 1.16 g pentahydrate form of CZ along with 0.2 g sodium carbonate as powder for injection solution.

## 3. Results and Discussion

### 3.1. Electrochemical Behavior of CZ

Studies on the possibility to use CZ as additive (levelling agent) in copper and nickel electrodeposition started with its electrochemical behavior, characterized by cyclic voltammetry recorded on platinum electrode in 0.5 mol L^−1^ H_2_SO_4_ and BS, in a wide potential range. Concerning the nature of acids and their concentrations, as well as the pH, both of the electrolyte solutions used are similar with those used at industrial level in galvanotechnics: 0.5 mol L^−1^ H_2_SO_4_ for copper electrodeposition from acid baths (pH = 0–1), respectively 30 g L^−1^ H_3_BO_3_ for nickel electrodeposition from Watts baths (pH = 3.5–4.5).

**Figure 2:**
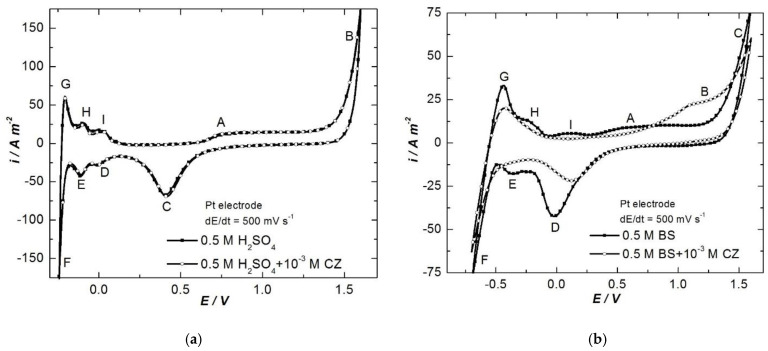
CVs on platinum electrode in 0.5 mol L^−1^ H_2_SO_4_ (**a**) and BS (**b**) without and with 10^−3^ mol L^−1^ CZ, at 500 mV s^−1^ scan rate.

Figure 2 illustrates the CVs drawn on platinum electrode with 500 mV s^−1^ scan rate in 0.5 mol L^−1^ H_2_SO_4_ (Figure 2a) and BS (Figure 2b).

Analyzing the curves from Figure 2a, it is observed that in strong acid media (H_2_SO_4_), CZ does not undergo electrochemical reactions at either the anode or cathode. At high scan rate, peaks and waves assigned to the superficial oxidation of platinum substrate A (about +0.75 V) and oxygen evolution B (about +1.50 V) can be distinguished (at anodic polarization), as well as its reduction peak C (approx. +0.40 V), followed by the generation of adsorbed hydrogen atoms (H_ads_) D (about 0 V), adsorbed hydrogen molecules (H_2ads_) E (approx. −0.15 V), and H_2_ evolution F (about −0.25 V), and their oxidation correspondents (G, H, I), when the potential is shifted towards positive values.

However, in weak acid media (H_3_BO_3_), the electrochemical behavior of CZ is different than in strong acid solution, most probably due to the change of the protonation degree of the active substance of the drug. On the curve drawn in BS (Figure 2b), an oxidation plateau (B) is observed around +0.75–+1.25 V, associated with CZ oxidation to various products.

**Figure 3:**
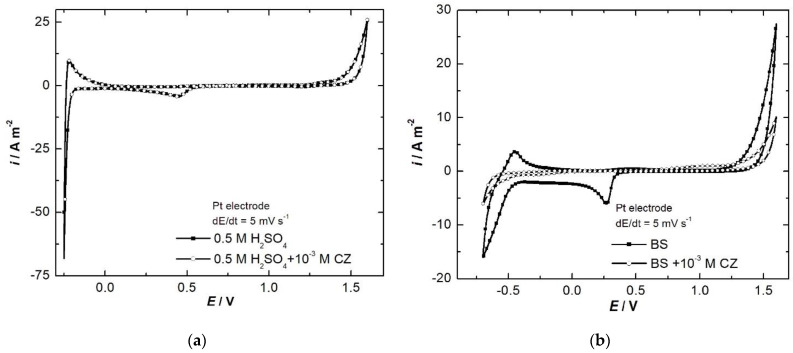
CVs (cycle 3) on platinum electrode 0.5 mol L^−1^ H_2_SO_4_ (**a**) and 0.5 mol L^−1^ Na_2_SO_4_ + 30 g L^−1^ H_3_BO_3_ (BS) (**b**) without and with 10^−3^ mol L^−1^ CZ, at 5 mV s^−1^ scan rate.

At low scan rate (5 mV s^−1^) (Figure 3) on the CVs recorded in H_2_SO_4_, only O_2_ evolution, O_2ads_ reduction, H_2_ evolution and H_2ads_ oxidation peaks and waves can be observed. In BS, in addition to these, the CZ oxidation wave can be seen.

Each wave’s intensity of oxygen and hydrogen evolution reaction diminishes, simultaneously with the increase of the overpotentials, due to the inhibitory effect of CZ and its oxidation products, which are adsorbed onto the electrode surface.

Based on the above presented CVs, it can be stated that in copper acid baths, CZ will not undergo any transformation at the electrodes; consequently, its concentration will remain almost constant during copper electroplating for long periods. In addition, CZ will not degrade, even in BS, since during anodic ionization of nickel the polarization is not pronounced enough to oxidize the organic compound.

### 3.2. Inhibitory Effect of CZ on Copper and Nickel Electrodeposition

In order to determine the kinetic parameters of copper and nickel electrodeposition, LVs have been recorded with low scan rate (2 mV s^−1^) on copper and nickel electrodes in solutions containing 5 g L^−1^ metal ions in 0.5 mol L^−1^ H_2_SO_4_ for copper bath and in 30 g L^−1^ H_3_BO_3_ for nickel bath. Both the influence of CZ added in the electrolyte solution and the temperature effect on these processes have been studied.

**Figure 4:**
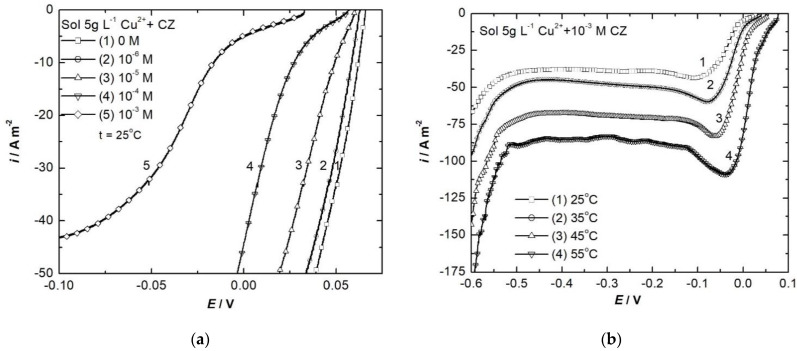
LVs recorded for copper electrodeposition without and with different concentrations of CZ at 25 °C (**a**) and with 10^−3^ mol L^−1^ CZ at different temperatures (**b**), 2 mV s^−1^ scan rate.

**Figure 5:**
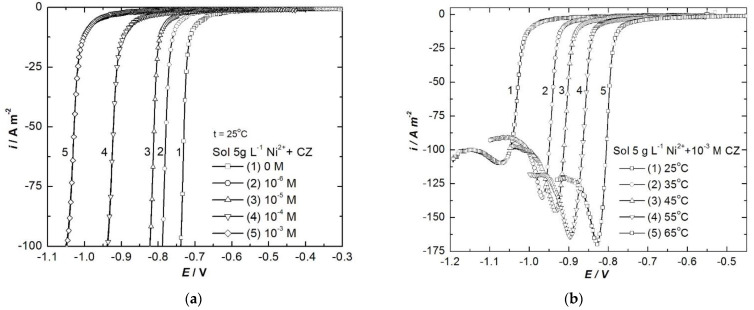
LVs recorded for nickel electrodeposition without and with different concentrations of CZ at 25 °C (**a**) and with 10^−3^ mol L^−1^ CZ at different temperatures (**b**), 2 mV s^−1^ scan rate.

LVs (Figure 4 and Figure 5) and correspondent Tafel plots [36] (Figures S1 and S2 presented in Supplementary Materials) have been drawn for Cu and for Ni electrode respectively in electrolyte solutions without and with different concentrations of CZ (between 10^−6^ and 10^−3^ mol L^−1^).

Knowing the beneficial effect of temperature on the electrodeposition mechanisms of both metals, as well as on the quality of the obtained metallic deposits, LVs were recorded in 25–55 °C temperature range for copper and 25–65 °C for nickel electrodeposition. Figure 4b presents the LVs plotted in 5 g L^−1^ Cu^2+^ and Figure 5b the ones in 5 g L^−1^ Ni^2+^, both of them with addition of 10^−3^ mol L^−1^ CZ as additive.

The LVs show that there are not additional peaks and waves in the presence of CZ, indicating the organic molecules are not implicated in any reduction processes; they are stable and the only phenomenon occurring at the interface is their adsorption onto the metal surface, thus inhibiting the metal deposition. This is also supported by the negative shift of about 100 mV for copper electrodeposition potential and 300 mV for nickel.

The kinetic parameters for copper electrodeposition are shown in Table 1 and for nickel electrodeposition in Table 2.

The cathodic charge transfer coefficient 1 − α is strongly influenced by the concentration of CZ in the electrolyte solution. It can be observed that increasing CZ concentration in the solution leads to 1 − α decrease because, according to Bockris et al.’s [37] considerations, the organic molecules are adsorbed at the metal/electrolyte solution interface, which means the reaction plane is shifted towards the bulk of the electrolyte solution. As can be observed from the LVs, this is equivalent with the decrease of the reaction rate, respective of the net density current passing through the electrode.

If temperature rises, the reverse phenomenon is observed; 1 − α increases since the thermal movement is enhanced and consequently Cu^2+^ ions can move closer to the metal surface, resulting in the intensification of the deposition process.

For the exchange current density *i_o_*, appreciable values are obtained in accordance with literature data reported by Farndon et al. [38] and Wan et al. [39]. The addition of CZ in the electrolyte solution leads to a small diminution of the exchange current density, which demonstrates the slight inhibitory effect of the organic compound on copper electrodeposition. It is worth noting that according with Butler–Volmer relation, the addition of CZ produces an inhibition phenomenon, modifying the kinetic parameters of this process in the direction in which the net current density through the interface is diminished. As expected, the temperature rise involves the increase of exchange current density because the activation energy of the cathodic process decreases.

CZ addition in the electrolyte solution has similar effect in nickel electrodeposition, but it is far more sensitive. Thus, at 25 °C, the exchange current density decreases more than 10^4^ times when 10^−3^ mol L^−1^ CZ is added in the electrolyte solution. However, the exchange current density values are smaller for nickel than copper deposition and nickel electrodeposition overpotential is higher than for copper. Furthermore, activation energy for nickel deposition is also larger.

In the case of copper electrodeposition, unusually high values are obtained for 1 − α since the Cu^2+^ reduction process occurs at high speed, and it is not controlled by the charge transfer process alone. On the other hand, because it is a fast process, the stationary state is not reached even at very low scan rates.

In the ideal case, when the electron transfer is the limiting process, the charge transfer resistance is expressed by Equation (1) [40]:

(1) $$R_{\mathrm{ct}}=\frac{\mathrm{RT}}{\left(1-\alpha \right){\mathrm{Fi}}_{o}}e^{\frac{\left(1-\alpha \right)F}{\mathrm{RT}}\eta }$$

where *R* is gas constant, *T*–thermodynamic temperature, (1 − α)–cathodic transfer coefficient, *F*–Faraday’s number, *η*–overpotential.

Knowing the exchange current density values within a limited temperature range, the activation energy for both copper and nickel electrodeposition processes has been calculated based on lg |*i_o_*| = *f* (*T*^−1^) dependence [Arrhenius plot given by Equation (2)] [41]:

(2) $$E_{a}=-2.303\left(\frac{\partial i_{o}}{\partial T^{-1}}\right)$$

**Figure 6:**
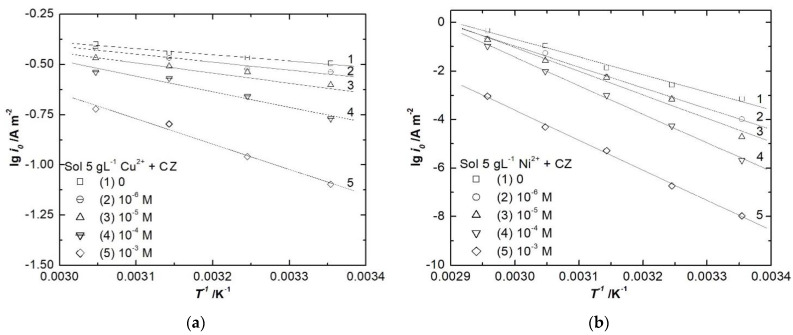
Arrhenius plots for copper (**a**) and nickel (**b**) deposition without and with different concentrations of CZ.

Analyzing Figure 6, it can be observed that the two electrochemical processes are more sensitive to CZ addition at low temperatures (25, 35 °C). At higher temperatures, the thermal movement increases, diminishing the organic compound inhibitory effect. The calculated values of activation energy are depicted in Table 3.

Conclusions obtained from the linear voltammetry data are confirmed by the activation energy values. When CZ is added in the copper and nickel baths, the activation energy increases proportionally with its concentration, therefore CZ exhibits an inhibitory effect for both electrochemical processes. Generally, it can be stated that copper electrodeposition occurs at a lower overpotential than for nickel, regardless of CZ addition in the electrolyte solutions.

**Figure 7:**
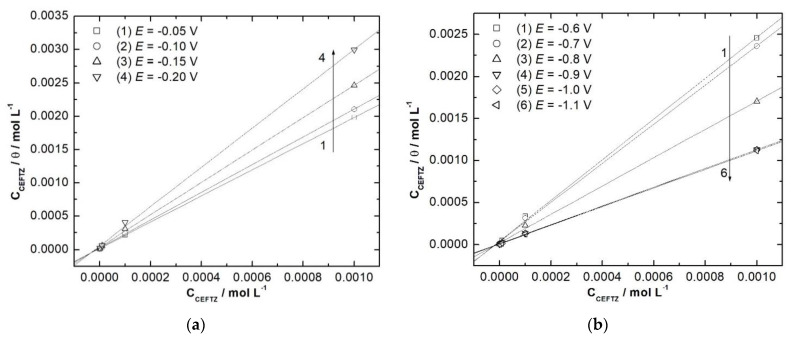
Langmuir isotherms for copper (**a**) and nickel electrodeposition (**b**) at different potential values.

Information about how the inhibitor acts on the electrodeposition processes has been found based on the adsorption isotherms drawn using the data provided by EIS (Figure 7). Given the chemical structure of CZ, Langmuir isotherm–Equation (3)–is the best approach, according to Koryta et al. [42].

(3) $$\frac{C_{\mathrm{inh}}}{\theta }=\frac{1}{K_{\mathrm{ads}}}+C_{\mathrm{inh}}$$

where *C*_inh_–CZ concentration added in the electrolyte solution (mol L^−1^); *θ*–coverage degree of the electrode; *K*_ads_–adsorption constant.

Representing the linear dependence between *C*_inh_/*θ* and *C*_inh_ (Figure 7), from abscissa values the adsorption constant K_ads_ is determined. Further, standard Gibbs energy ${\Delta G}_{\mathrm{ads}}^{o}$ is calculated using the Equation (4) [43]:

(4) $${\Delta G}_{\mathrm{ads}}^{o}=-\mathrm{RT}\ln\left(55.5K_{\mathrm{ads}}\right)$$

It is well known that the value of Gibbs free energy gives information about the nature of interactions between the substrate and the adsorbed compound. Amin and Ibrahim [44] suggested that values close to −20 kJ mol^−1^ show a physical bound between the substrate and the organic compound, while −40 kJ mol^−1^ indicates a chemical one (chemisorption). Values obtained for copper and nickel electrodeposition are presented in Table 4.

Both in nickel and copper cases, ${\Delta G}_{\mathrm{ads}}^{o}$ values are close to −40 kJ mol^−1^ which indicates that during the electrodeposition, CZ is chemically adsorbed onto the metal surface.

### 3.3. Morphological Characterization

**Figure 8:**
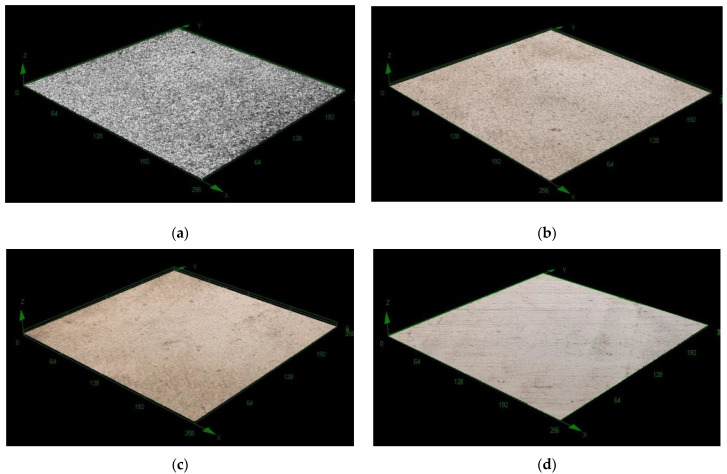
Images for copper (**a**,**b**) and nickel electrodeposition (**c**,**d**) without/with 10^−4^ mol L^−1^ CZ, magnification: 50×.

To highlight CZ influence on the morphology of the metallic layers obtained by electrodeposition, copper and nickel industrial bath were used. In those baths, CZ was added, and the morphologies of the layers deposited without and with CZ were compared. The layers have been deposited using the following parameters (Table 5):

Figure 8 illustrates the images obtained by optical microscopy for copper layers, and for nickel respectively. For both metals, there are presented comparatively the deposits obtained without and with CZ addition.

It can be seen that in the absence of CZ a rough structure is obtained, while when a small amount (10^−4^ mol L^−1^) of CZ is added in the electrodeposition bath, the layers have a bright aspect in both copper and nickel cases. For all samples, 5 different areas were investigated on each sample and the average values for the mean roughness parameters (*R*_a_) were: for copper deposition (without CZ–10.3 μm and with CZ–1.84 μm) and for nickel deposition (without CZ–1.02 μm and with CZ–0.128 μm). If a higher concentration of CZ is added, the layers are qualitative inadequate, having a matte look.

## 4. Conclusions

In this paper, the possibility of using CZ, the active substance from Ceftamil^®^ pharmaceutical drug as an additive in copper and nickel acid baths was studied.

Cyclic voltammetry was conducted in order to emphasize the electrochemical behavior and stability of CZ in acid media, similar to the galvanic baths used at commercial level. It was found that in strong acid media (H_2_SO_4_), CZ does not undergo electrochemical transformations, while in weak acid media (H_3_BO_3_), at advanced anodic polarization, it oxidizes to different compounds. However, CZ can be used in both baths since the polarization is not pronounced enough during the metal deposition to oxidize the compound.

LV studies proved that CZ acts as an inhibitor on the studied processes, the copper and nickel characteristic overpotentials being shifted towards more negative values, proportional with the amount of CZ added in the electrolyte solution. Based on the LVs, from Tafel plots, kinetic parameters (charge transfer coefficient 1-*α* and the exchange current density *i_o_*) for copper and nickel deposition have been calculated. In nickel case, the increase of 1-*α* and the advanced decrease of *i_o_* (10^4^ times) are observed with the increase of CZ concentration in the electrolyte solution, meaning the reaction plane is shifted toward the solution bulk due to the partially block of the electrode surface with CZ large molecules, inhibiting thus the deposition process. In copper case, a similar but not as advanced effect as for nickel is noticed. With the increase of temperature from 25 to 65 °C, the reverse phenomenon is observed, the processes being accelerated as a result of the thermal movement intensification.

As well, the activation energy values, obtained from Arrhenius plots, confirm the inhibitory effect of CZ, *E*_a_ increasing with the increase of CZ amount added in the electrolyte solution.

Forwards, from Langmuir isotherms, Gibbs free energy was approximated. The values are close to −40 kJ mol^−1^, suggesting a chemical adsorption of CZ onto the metallic electrode surface.

The morphology of the copper and nickel deposited layers showed CZ has a levelling effect when it is used as additive in small concentration; the proper amount to be used is about 10^−4^ mol L^−1^, at higher concentrations, the layers are qualitatively inadequate.

## Figures and Tables

**Table 1:** Kinetic parameters for copper deposition from 5 g L^−1^ Cu^2+^ without and with different concentrations of CZ, in 25–55 °C temperature range.

CZ Conc. (mol L^−1^)	*t* (°C)	1 − *α*	*i_o_* (A m^−2^)
0	25	0.75	0.32
	35	0.82	0.34
	45	0.88	0.36
	55	0.91	0.40
10^−6^	25	0.73	0.29
	35	0.78	0.30
	45	0.86	0.34
	55	0.89	0.38
10^−5^	25	0.67	0.24
	35	0.74	0.27
	45	0.82	0.31
	55	0.87	0.34
10^−4^	25	0.64	0.17
	35	0.75	0.22
	45	0.78	0.27
	55	0.83	0.29
10^−3^	25	0.39	0.08
	35	0.47	0.11
	45	0.53	0.16
	55	0.55	0.19

**Table 2:** Kinetic parameters for nickel deposition from 5 g L^−1^ Ni^2+^ without and with different concentrations of CZ, in 25–65 °C temperature range.

CZ Conc. (mol L^−1^)	*t* (°C)	1 − *α*	*i_o_* (A m^−2^)
0	25	0.53	7.14·10^−4^
	35	0.57	2.72·10^−3^
	45	0.61	1.37·10^−2^
	55	0.67	1.11·10^−1^
	65	0.74	4.60·10^−1^
10^−6^	25	0.52	1.04·10^−4^
	35	0.56	7.42·10^−4^
	45	0.60	5.87·10^−3^
	55	0.65	5.49·10^−2^
	65	0.69	2.15·10^−1^
10^−5^	25	0.50	1.93·10^−5^
	35	0.54	6.90·10^−4^
	45	0.58	5.44·10^−3^
	55	0.63	2.67·10^−2^
	65	0.67	1.92·10^−1^
10^−4^	25	0.46	2.08·10^−6^
	35	0.51	5.52·10^−5^
	45	0.57	1.01·10^−3^
	55	0.62	9.91·10^−3^
	65	0.65	1.10·10^−1^
	25	0.44	1.07·10^−8^
	35	0.49	1.84·10^−7^
10^−3^	45	0.54	5.27·10^−6^
	55	0.60	4.98·10^−5^
	65	0.63	9.20·10^−4^

**Table 3:** Activation energy values for copper and nickel electrodeposition without and with different concentrations of CZ.

CZ Conc. (mol L^−1^)	Cu/Cu^2+^	Ni/Ni^2+^
	*E_a_* (kJ mol^−1^)	
0	5.87	139
10^−6^	7.59	164
10^−5^	9.64	186
10^−4^	14.8	225
10^−3^	24.2	238

**Table 4:** Gibbs free energy for nickel and copper deposition at different potentials.

Process	E (V)	${\Delta G}_{\mathrm{ads}}^{o}$ (kJ mol^−1^)
Nickel electrodeposition	−0.6	−35.0
	−0.7	−35.3
	−0.8	−36.6
	−0.9	−38.0
	−1.0	−38.9
	−1.1	−39.7
Copper electrodeposition	−0.05	−37.1
	−0.10	−36.2
	−0.15	−35.4
	−0.20	−34.2

**Table 5:** Electrodeposition parameters.

Copper deposition	Substrate	Cu
	Electrodeposition bath	250 g L^−1^ CuSO_4_·5H_2_O 0.5 mol L^−1^ H_2_SO_4_ without/with 10^−4^ mol L^−1^ CZ
	Current density	100 A m^−2^
	Time	15 min
	Temperature	25 °C
Nickel deposition	Substrate	Cu
	Electrodeposition bath	300 g L^−1^ NiSO_4_·7H_2_O 45 g L^−1^ NiCl_2_·6H_2_O 30 g L^−1^ H_3_BO_3_ without/with 10^−4^ mol L^−1^ CZ
	Current density	200 A m^−2^
	Time	10 min
	Temperature	25 °C

## Data Availability

Data sharing is not applicable to this article.

## Supplementary Materials

The following are available online at https://www.mdpi.com/article/10.3390/ijerph18189476/s1, Figure S1: Tafel plots for copper electrodeposition without and with different concentrations of CZ at 25 °C (a) and with 10^−3^ mol L^−1^ CZ at different temperatures (b), 2 mV s^−1^ scan rate, Figure S2: Tafel plots for nickel electrodeposition without and with different concentrations of CZ at 25 °C (a) and with 10^−3^ mol L^−1^ CZ at different temperatures (b), 2 mV s^−1^ scan rate, Figure S3: Nyquist (a) and Bode plots (b) recorded on nickel electrode in 5 g L^−1^ Ni^2+^ different CZ concentrations at E = −0.80 V, Figure S4: Nyquist (a) and Bode plots (b) recorded on nickel electrode in 5 g L^−1^ Ni^2+^ different CZ concentrations at E = −0.80 V, Figure S5: Equivalent electrical circuit for modelling nickel (a) and copper (b) electrodeposition, Table S1: Calculated data of the circuit elements and experimental errors (between brackets, %) for nickel electrodeposition, Table S2: Calculated data of the circuit elements and experimental errors (between brackets, %) for copper electrodeposition.
