# Supplementary material for: Recycling of Expired Ceftamil Drug as Additive in the Copper and Nickel Electrodeposition from Acid Baths
Source: Int J Environ Res Public Health. 2021 Sep 8;18(18):9476. doi: 10.3390/ijerph18189476 (PMC8467844; doi:10.3390/ijerph18189476)
Supplement: Supplementary file 1 [file ijerph-18-09476-s001.zip › ijerph-1318649-supplementary.pdf]

International Journal of
Environmental Research and Public Health

MDPI

Supplementary Material

Tafel plots

Tafel plots were drawn using the dependence i = f (E) of the linear voltammograms in the domain where the charge transfer is the limiting process (Figure S1 and Figure S2). According to Butler – Volmer equation applied in the high overpotentials region, the charge transfer coefficient 1-α has been calculated from the Tafel slopes values b (equation S1) and the exchange current density i_o from the abscissa values a (equation S2) [36].

$$b = -\frac{2.303RT}{(1-\alpha)zF} \tag{S1}$$

$$a = \frac{2.303RT}{(1-\alpha)zF}\lg i_o \tag{S2}$$

where: R is the gas constant (J mol^{-1} K^{-1}); T – thermodynamic temperature (K); 1-α – charge transfer coefficient; z – elementary charge number; F – Faraday's number (C mol^{-1}); i_o – exchange current density (A m^{-2}).

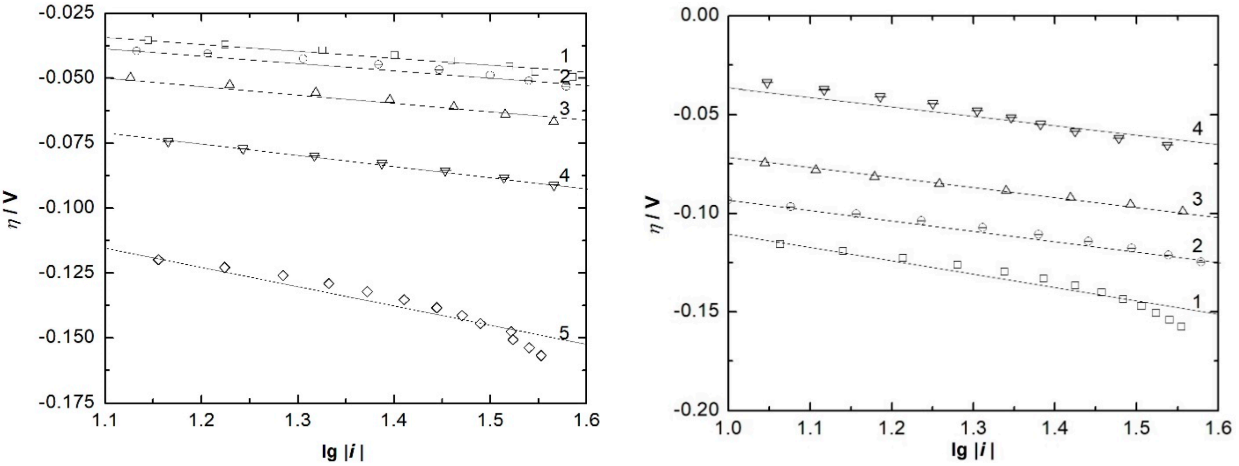

(a) (b)

Figure S1. Tafel plots for copper electrodeposition without and with different concentrations of CZ at 25°C (a) and with 10^{-3} mol L^{-1} CZ at different temperatures (b), 2 mV s^{-1} scan rate.

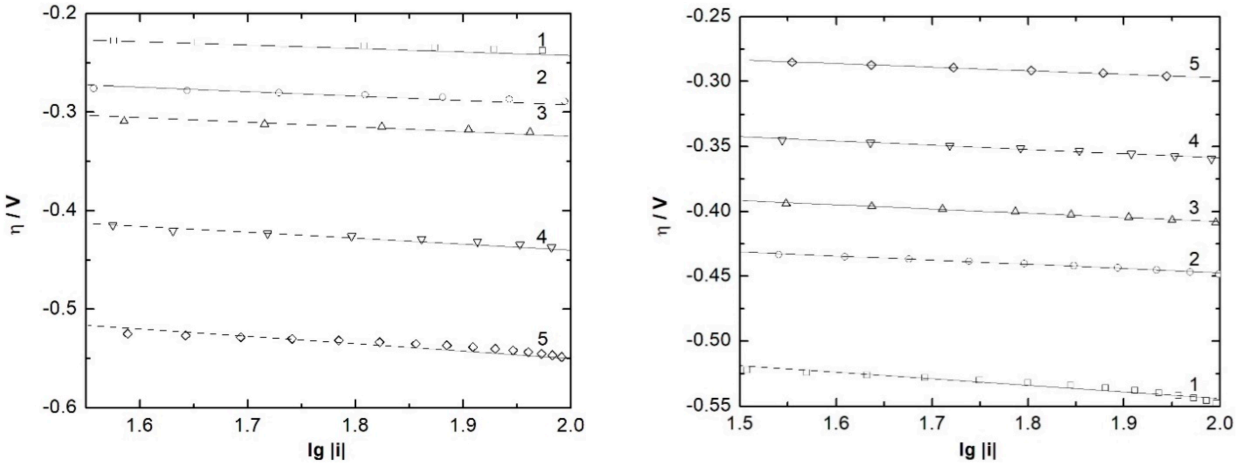

(a) (b)

Figure S2. Tafel plots for nickel electrodeposition without and with different concentrations of CZ at 25°C (a) and with 10^{-3} mol L^{-1} CZ at different temperatures (b), 2 mV s^{-1} scan rate.

Electrochemical impedance spectroscopy

For more precise characterization of the processes occurring at the interface, the charge transfer resistance R_{ct} and double layer capacity C_{dl} have been evaluated from EIS measurements. Furthermore, the surface coverage degree θ has been calculated using equation (S3).

$$\theta = \frac{R_{ct}^{inh} - R_{ct}}{R_{ct}^{inh}} \quad \text{(S3)}$$

where R_{ct} and R_{ct}^{inh} are the charge transfer resistances in solutions without and with CZ addition respectively.

Relying on the previously presented LVs, an optimal potential range was selected in which nickel deposition is the only process that occurs at the interface (-0.6 ÷ -1.1 V).

In Figure S3, EIS results expressed as Nyquist plots for nickel electrodeposition from 5 g L^{-1} Ni^{2+} solution in the absence and presence of 10^{-6} ÷ 10^{-3} mol L^{-1} CZ, at -0.8 V are presented. In Figure S4, the same diagrams for copper electroplating from 5 g L^{-1} Cu^{2+} with 10^{-4} mol L^{-1} CZ, at different potentials are depicted.

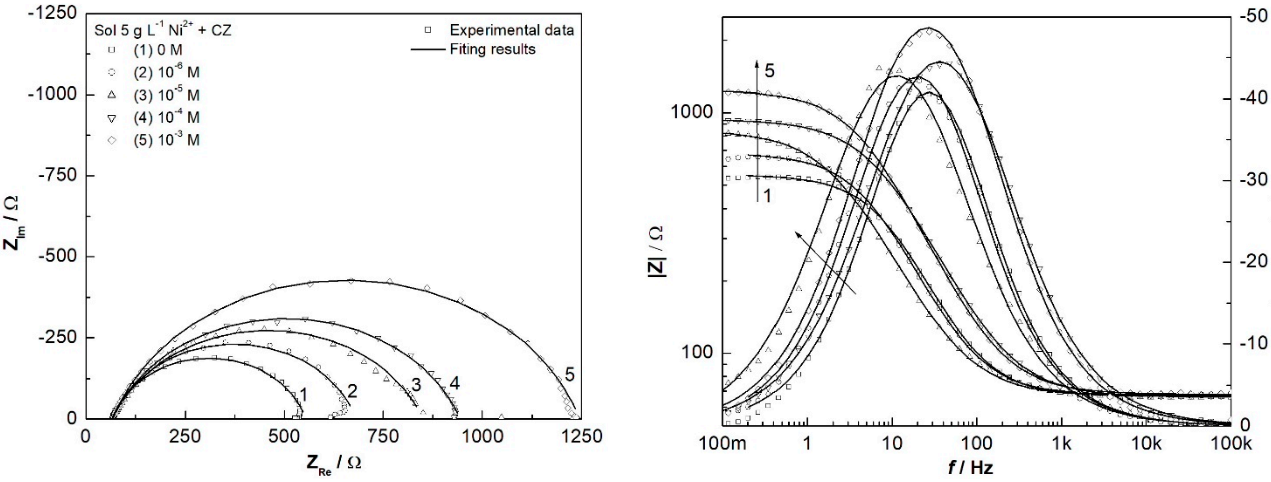

(a) (b)

Figure S3. Nyquist (a) and Bode plots (b) recorded on nickel electrode in 5 g L^{-1} Ni^{2+} different CZ concentrations at E= -0.80 V.

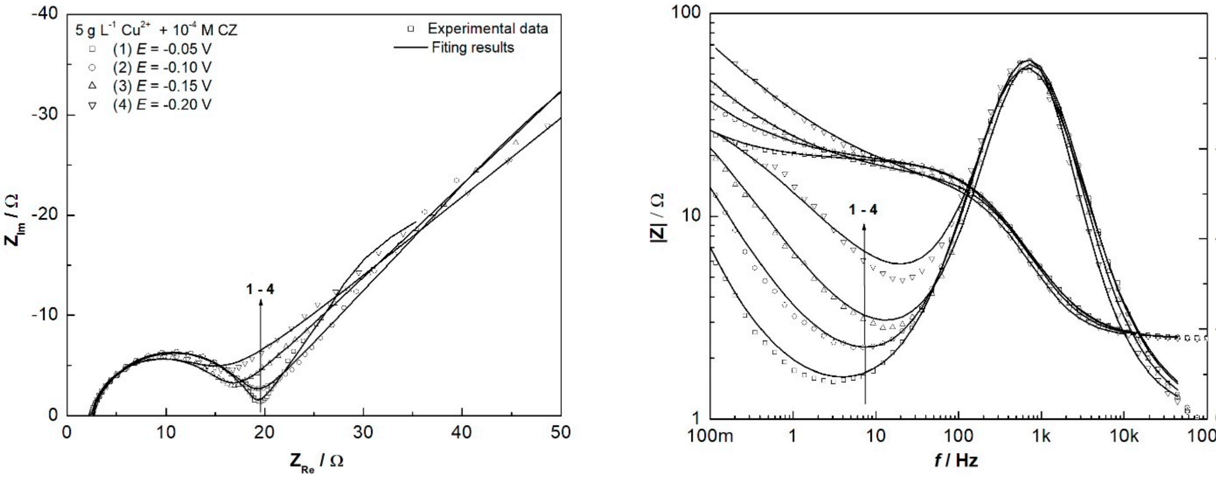

(a) (b)

Figure S4. Nyquist (a) and Bode plots (b) recorded on nickel electrode in 5 g L^{-1} Ni^{2+} different CZ concentrations at E= -0.80 V.

In the case of nickel, the shape of Nyquist spectra (Figure S4), as a slightly suppressed semicircle, indicates an electron transfer limiting process, characterised by the charge transfer resistance (R_{ct}). An obvious dependence between diameters of the semicircles and CZ concentration added in the electrolyte solution has been observed. The enlargement

of the diameters with the increase of the additive concentration proves its inhibitory effect on the nickel deposition process. Nyquist spectra for copper electrodeposition consist of a semicircle characteristic to Cu^{2+} reduction at the interface, followed by the component owed to the Cu^{2+} ions diffusion from the bulk solution to the interface.

Experimental data have been fitted to the electrical equivalent circuits (EEC) presented in Figure S5, using a complex non-linear least square procedure.

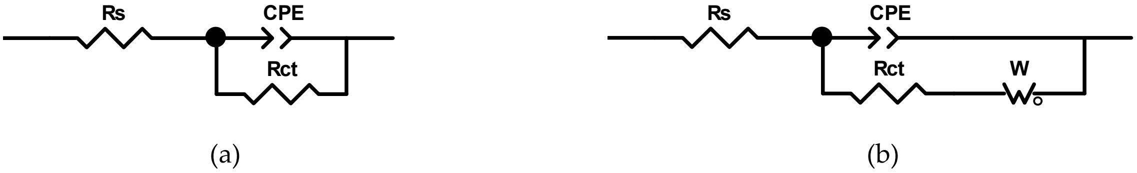

Figure S5. Equivalent electrical circuit for modelling nickel (a) and copper (b) electrodeposition.

First EEC, used to analyze the impedance data for nickel deposition, consisted of an ohmic resistance (R_s) connected in series with a parallel connection between the charge transfer resistance (R_{ct}) and double layer capacity (DLC). The second one, used by Vaduva et al. [45] for copper deposition, is the Randles circuit for semi-infinite linear diffusion R_s (CPE (R_{ct}, W_s)). It consists in a solution resistance R_s which represents the uncompensated solution resistance, bound in series with a parallel connection between a constant phase element (*CPE*) and a charge transfer resistance R_{ct} in series with a Warburg element (W_s) characteristic of diffusion. In the real electrochemical systems, *CPE* element characterizes more precisely double layer capacity (C_{dl}), thus replacing the ideal capacitor (*C*). *CPE* impedance can be describe by the equation S4 [42]:

$$CPE = \frac{1}{T(j\omega)^n} \tag{S4}$$

where *T* is *CPE* capacity element, ω – angular frequency, *j* – imaginary vector unit and *n* – CPE exponent.

If $n = 1$, then *CPE* element act as pure capacitor (C_{dl}) and when n = 0.5 it means a pure diffusion. The Warburg element, typical to describe the mass diffusion process, has a similar expression for impedance as *CPE*, but with three components W_R - resistive part, W_T – inductive part and W_P – exponent.

Calculated data of the circuit elements on Ni in 5 g L^{-1} Ni^{2+} without and with various concentrations of CZ and surface coverage degree values are presented in Table S1.

Table S1. Calculated data of the circuit elements and experimental errors (between brackets, %) for nickel electrodeposition.

E (*V*)	*CZ conc.* (mol L^{-1})	R_s (Ω cm^2)	$T\cdot10^5$ ($F\ cm^{-2}\ s^{n-1}$)	*n*	R_{ct} (Ω cm^2)	$C_{dl}\cdot10^5$ ($F\ cm^{-2}$)	$Chi^2\cdot10^3$	θ (%)
-0.6	0	66.6 (0.40)	16.7 (0.80)	0.79 (0.29)	4937 (0.78)	15.9	2.8	-
	10^{-6}	66.9 (0.47)	12.1 (0.98)	0.77 (0.31)	5485 (0.75)	10.8	2.5	9.9
	10^{-5}	66.1 (0.54)	8.35 (1.16)	0.76 (0.34)	6100 (0.78)	7.75	3.6	19.1
	10^{-4}	66.2 (0.38)	7.78 (0.78)	0.76 (0.26)	7039 (0.55)	6.44	1.7	29.9
	10^{-3}	66.3 (0.31)	7.11 (0.61)	0.75 (0.19)	8325 (0.50)	5.89	1.3	40.7
-0.7	0	65.9 (0.24)	10.8 (0.91)	0.80 (0.20)	1789 (0.29)	7.16	1.1	-
	10^{-6}	67.1 (0.50)	9.87 (1.50)	0.78 (0.40)	2043 (0.63)	6.31	3.4	12.4
	10^{-5}	66.4 (0.36)	8.85 (0.96)	0.77 (0.31)	2253 (0.56)	5.54	2.5	20.6
	10^{-4}	65.9 (0.23)	7.35 (0.69)	0.75 (0.18)	2632 (0.28)	4.29	0.7	32.0
	10^{-3}	67.3 (0.37)	6.78 (0.97)	0.75 (0.27)	3107 (0.49)	4.01	1.7	42.4
-0.8	0	66.7 (0.16)	9.17 (1.00)	0.83 (0.22)	487 (0.28)	4.94	0.6	-
	10^{-6}	67.2 (0.23)	8.87 (1.26)	0.82 (0.30)	634 (0.43)	4.69	1.1	23.1
	10^{-5}	66.3 (0.31)	7.85 (1.34)	0.79 (0.37)	785 (0.53)	3.80	1.9	36.9
	10^{-4}	65.8 (0.15)	6.65 (0.69)	0.78 (0.16)	879 (0.19)	2.84	0.3	44.6
	10^{-3}	67.6 (0.19)	6.12 (0.81)	0.76 (0.19)	1178 (0.26)	2.67	0.6	58.7
-0.9	0	65.9 (0.12)	8.05 (1.27)	0.90 (0.61)	43.8 (0.38)	4.31	1.7	-
	10^{-6}	68.1 (0.12)	6.89 (1.08)	0.88 (0.52)	58.5 (0.42)	3.22	0.8	25.2

	10^{-5}	67.8 (0.13)	6.45 (1.41)	0.87 (0.44)	76.6 (0.40)	2.80	0.7	42.8
	10^{-4}	69.3 (0.21)	5.79 (1.16)	0.86 (0.44)	175 (0.48)	2.69	1.0	74.9
	10^{-3}	66.5 (0.27)	5.03 (1.16)	0.83 (0.39)	395 (0.39)	2.20	0.9	88.7
-1.0	0	66.4 (0.10)	6.04 (2.8)	0.95 (0.73)	21.1 (0.50)	4.41	1.6	-
	10^{-6}	68.5 (0.10)	5.81 (2.9)	0.94 (0.75)	29.3 (0.60)	3.89	0.8	28.1
	10^{-5}	68.9 (0.13)	5.54 (2.3)	0.93 (0.82)	49.1 (0.68)	3.46	1.2	57.1
	10^{-4}	70.1 (0.15)	5.19 (1.6)	0.89 (0.54)	91.6 (0.50)	2.77	0.8	77.0
	10^{-3}	68.1 (0.34)	5.07 (2.4)	0.84 (0.79)	194 (0.68)	2.12	1.9	89.1
-1.1	0	67.1 (0.08)	5.71 (2.05)	0.97 (0.86)	10.2 (0.61)	4.76	1.3	-
	10^{-6}	69.0 (0.14)	5.54 (2.61)	0.94 (1.35)	14.9 (0.98)	3.67	1.5	32.0
	10^{-5}	68.2 (0.09)	5.37 (1.62)	0.92 (0.82)	17.6 (0.74)	2.88	0.5	62.1
	10^{-4}	67.5 (0.16)	5.14 (1.89)	0.90 (0.93)	51.2 (0.90)	2.67	0.9	80.2
	10^{-3}	70.0 (0.11)	4.90 (2.36)	0.85 (1.02)	97.1 (1.45)	1.95	1.4	89.7

Analyzing data from table S1 it can be noted that the charge transfer resistance R_{ct} is strongly influenced by the presence of CZ in the electrolyte solution. R_{ct} increases proportionally with the concentration of organic compound, which confirms the inhibitory effect of CZ on nickel electrodeposition. Also, R_{ct} decreases according to the Butler – Volmer equation when the cathodic polarization is raised.

Since the quantity of CZ added is low, as expected, the solution resistance R_s does not undergo significant changes. However, the organic compound addition strongly influences the double layer capacity C_{dl} due to its adsorption on the cathode which results in the increase of CZ amount in the vicinity of the electrode, partially blocking it. Consequently, inner and outer Helmholtz planes are shifted towards the solution bulk, equivalent with increasing distance between the plates of a capacitor [40].

On nickel, the electrode process is controlled by the charge transfer step, so it can be considered that the C_{dl} is not sensitive to the change of the electrode potential. In other words, Ni^{2+} particles diffuse or migrate faster than they are reduced to the interface. Since the capacity of a condenser is inversely proportional with the voltage between its plates, increasing the electrode potential will lead to the decrease of C_{dl}.

Coverage degree of the electrode surface with organic molecules also indicates that CZ is an adsorption inhibitor, higher values being obtained with the increase of CZ concentration in the electrolyte solution.

Data on the copper cathodic deposition (Table S2) show that, similar with the nickel deposition, R_{ct} is sensitive to the presence of CZ into the electrolyte solution. CZ adsorption on the electrode surface, competitive with Cu^{2+} ions reduction, leads to the partial block of the electrode and so to the increase of the polarization resistance R_{ct}. Surely, also in this case, R_s is slightly influenced by CZ addition in the solution.

Unlike nickel deposition, copper deposition is controlled by both charge transfer and mass transport step of electroactive particles Cu^{2+} from the solution bulk towards the metal/electrolyte solution interface, as it was seen from Nyquist diagrams (Figure S4a). Increasing the overpotential of copper deposition, the electric field in the solution near the metal surface is also increased, which means the number of Cu^{2+} ions from the electrochemical double layer becomes higher because the migration process is intensified. Therefore, C_{dl} will increase. This behaviour will compensate and overcome the C_{dl} decrease due to the increase of the electrode potential, especially at low overpotentials. In fact, the coverage degree of the copper cathode with CZ compound also decreases when the polarization is increased, some of the organic molecules being replaced with Cu^{2+} ions.

Warburg resistance W_R provides information about how the mass transport of Cu^{2+} electroactive species from the solution bulk towards the electrode is influenced by the presence of the organic compound into the electrolyte solution. A high coverage degree of the cathode with CEFTZ involves a high W_R. As expected, increasing the polarization determines the decrease of W_R since the electric field of the solution adjacent to the electrode surface is intensified.

Table S2. Calculated data of the circuit elements and experimental errors (between brackets, %) for copper electrodeposition.

E (*V*)	*CEFTZ conc.* (*mol L*$^{-1}$)	R_s (Ω *cm*^{2})	$T \cdot 10^5$ (*F cm*$^{-2}$ *s*$^{n-1}$)	*n*	R_{ct} (Ω *cm*^{2})	W_R (Ω *cm*^{2})	W_T (*H cm*^{2})	W_P	$C_{dl} \cdot 10^5$ (*F cm*$^{-2}$)	$Chi^2 \cdot 10^3$	θ (%)
-0.05	0	2.92 (0.20)	4.58 (2.50)	0.92 (0.28)	9.13 (0.68)	16.8 (5.81)	13.7 (1.60)	0.35 (3.12)	2.25	0.10	-
	10^{-6}	2.78 (0.20)	4.99 (2.23)	0.90 (0.26)	10.2 (0.86)	23.6 (3.98)	26.6 (1.18)	0.37 (1.76)	3.98	0.11	10.79
	10^{-5}	2.64 (0.25)	9.73 (2.48)	0.90 (0.42)	12.2 (1.28)	40.9 (1.94)	31.2 (5.83)	0.37 (1.01)	4.81	0.19	25.23
	10^{-4}	2.46 (0.59)	10.2 (3.80)	0.87 (0.60)	16.6 (0.79)	69.7 (5.68)	38.2 (1.23)	0.38 (1.82)	4.69	1.80	44.91
	10^{-3}	2.74 (1.09)	11.7 (4.14)	0.78 (0.93)	18.4 (1.59)	95.4 (7.10)	40.7 (1.27)	0.39 (1.53)	4.19	3.49	50.51
-0.10	0	2.92 (0.33)	4.66 (3.80)	0.93 (0.45)	8.43 (0.88)	81.1 (5.20)	10.6 (3.33)	0.36 (1.10)	2.50	0.28	-
	10^{-6}	2.79 (0.31)	4.42 (3.54)	0.91 (0.41)	9.32 (0.71)	86.3 (2.62)	22.7 (0.61)	0.37 (0.79)	2.13	0.24	9.55
	10^{-5}	2.65 (0.57)	8.99 (3.83)	0.91 (0.63)	10.4 (2.76)	105 (3.78)	26.0 (5.03)	0.38 (2.23)	4.48	0.94	19.24
	10^{-4}	2.47 (0.80)	11.5 (4.13)	0.87 (0.86)	14.3 (1.21)	156 (1.21)	32.3 (2.69)	0.41 (1.57)	4.56	2.03	41.05
	10^{-3}	2.65 (1.02)	13.1 (5.75)	0.81 (0.94)	16.1 (2.52)	197 (6.11)	38.8 (2.85)	0.43 (3.09)	5.19	3.12	47.51
-0.15	0	2.92 (0.58)	5.56 (1.91)	0.94 (0.22)	7.66 (0.88)	94.5 (1.13)	13.0 (2.62)	0.46 (1.13)	3.27	0.80	-
	10^{-6}	2.77 (0.18)	5.96 (2.35)	0.93 (0.27)	8.22 (0.50)	104 (5.30)	29.8 (2.00)	0.47 (0.50)	3.33	0.07	6.83
	10^{-5}	2.66 (0.66)	11.3 (2.99)	0.91 (0.33)	9.32 (2.25)	139 (1.62)	22.7 (4.15)	0.47 (1.45)	5.88	1.13	17.85
	10^{-4}	2.51 (0.73)	12.9 (3.48)	0.88 (0.66)	11.4 (1.61)	203 (1.91)	27.6 (4.32)	0.48 (1.34)	5.43	2.03	32.84
	10^{-3}	2.72 (0.82)	15.5 (4.95)	0.83 (0.89)	12.9 (2.44)	235 (1.18)	32.4 (2.67)	0.49 (1.63)	4.37	1.45	40.65
-0.20	0	2.93 (0.65)	5.32 (2.61)	0.96 (0.51)	7.23 (2.62)	134 (1.78)	6.45 (3.52)	0.47 (1.35)	3.72	0.99	-
	10^{-6}	2.80 (0.73)	5.22 (3.34)	0.93 (0.68)	7.52 (3.42)	168 (1.71)	18.0 (4.02)	0.48 (1.31)	2.92	2.17	3.90
	10^{-5}	2.63 (0.46)	12.0 (3.64)	0.92 (0.78)	8.58 (3.16)	197 (2.27)	20.5 (1.27)	0.48 (0.84)	6.77	0.55	15.74
	10^{-4}	2.53 (0.93)	13.4 (4.61)	0.90 (0.89)	9.59 (3.17)	275 (3.13)	24.2 (5.33)	0.49 (1.43)	6.61	2.43	24.65
	10^{-3}	2.73 (1.13)	17.5 (5.13)	0.86 (0.96)	10.8 (3.13)	329 (5.99)	28.3 (1.29)	0.50 (1.44)	6.28	1.46	33.36
